# Identification of a Novel 15q21.1 Microdeletion in a Family with Marfan Syndrome

**DOI:** 10.1155/2022/3556302

**Published:** 2022-04-05

**Authors:** Rencong Yang, Wu Zhang, Hua Lu, Jinlong Liu, Yu Xia, Shengjie Liao, Xiaohui Li, Xiaoshen Zhang, Xiaoping Fan, Chaojie Wang

**Affiliations:** ^1^Department of Cardiovascular Surgery, The First Affiliated Hospital, Jinan University, Guangzhou, China; ^2^Department of Cardiothoracic Surgery, The Second People's Hospital of Foshan, Foshan, China; ^3^Department of Thoracic and Cardiovascular Surgery, Shanghai Children's Medical Center, Shanghai Jiao Tong University School of Medicine, Shanghai, China; ^4^Department of Cardiovascular Surgery, Guangdong Provincial Hospital of Chinese Medicine, Guangzhou, China; ^5^The Second Affiliated Hospital of Guangzhou University of Chinese Medicine, Guangzhou, China

## Abstract

**Background:**

Marfan syndrome (MFS) is a connective tissue disease involving multiple systems, with thoracic aortic aneurysm (TAA) as the most common life-threatening manifestation.

**Method:**

A pedigree with TAA was investigated, and peripheral venous blood was extracted from six family members. After whole exome sequencing (WES) and chromosomal microarray analysis (CMA) in these individuals, bioinformatics and inheritance analyses were performed.

**Result:**

WES revealed a novel, small, 0.76 Mb microdeletion in 15q21.1, which cosegregated with the disease phenotype in the family and led to the haploinsufficiency of the fibrillin 1 (*FBN1*) gene, which is associated with MFS. This small copy number variant (CNV) was confirmed by CMA.

**Conclusion:**

Our study expands the phenotypic spectrum of the pathogenic CNV associated with MFS, thereby facilitating clinical genetic diagnosis and future genetic counseling for this family.

## 1. Introduction

Marfan syndrome (MFS; OMIM: 154700) is a rare but severe connective tissue disorder with a variable phenotypic spectrum that includes lesions of the cardiovascular system, skeletal system, ocular system, and pulmonary system. The estimated prevalence of MFS is one in 5000 to 20000 live births, and the incidence of classic MFS is about 2–3 cases per 10000 individuals/adults [1]. The estimated prevalence rate of MFS is 1–2 cases per 10000 individuals in China [2].

The classic clinical manifestations of MFS include thoracic aortic aneurysm (TAA), wrist and thumb sign, special facial features (dolichocephaly, malar hypoplasia, enophthalmos, retrognathia, and down-slanting palpebral fissures), and high myopia. When not all the abovementioned clinical manifestations appear but only some of them, a term atypical MFS is used. Generally, clinical symptoms appear between 20 and 40 years of age [3], and the condition of most patients deteriorates with age. Among all the clinical manifestations, TAA and subsequent aortic dissecting aneurysm (ADA) are the major causes of reduced life expectancy in MFS patients, which results in mortality as high as 70%–90% [4]. Moreover, patients usually do not feel any chest pain, or they only occasionally have mild chest pain; however, once the aneurysm breaks, patients feel acute chest pain, and the probability of sudden death is 25% [5]. Even after undergoing operations for TAA repair, nearly 45% of patients develop new cardiovascular problems in the following 10 years [6]. MFS is an important risk factor of sudden death, and the quality of life of patients is affected by MFS even if treated in time.

The treatment of MFS is mainly symptomatic, including medical conservative therapies and surgical treatment of TAA and dissection. The main strategy of medical treatment is to delay the course of disease and limit the expansion rate of aortic root. Beta blockers (*β*-blockers) can significantly reduce blood pressure and heart rate and protect blood vessels. Angiotensin-receptor blockers (ARBs) can reduce the levels of TGF-*β* and its receptors. *β*-Blockers or ARBs are generally used together at diagnosis or upon documentation of significant and/or progressive aortic dilatation to reduce hemodynamic stress on the aortic wall. Other antihypertensive agents can be used if *β*-blockers and ARBs are not tolerated. However, medical treatment cannot prevent the result of aortic aneurysm or dissection, which is why surgical intervention is essential. Surgical repair of the aorta is recommended when the maximum measurement of the aortic root approaches 5.0 cm in adults or older children; the rate of increase of the aortic root diameter approaches 0.5–1.0 cm per year; or there is progressive and severe aortic regurgitation. More important are early diagnosis and prevention of the disease, which can bring early intervention to patients and improve curative effect.

In the past, the revised diagnostic criteria for MFS (the International Nosology of Heritable Disorders of Connective Tissue Meeting in Berlin, 1986) were used as the first-tier diagnostic criteria for MFS [1]. The Ghent nosology criteria were established in 1996 [7] and revised in 2010 [8]. Compared with the Ghent nosology, the revised Ghent nosology can better identify and differentially diagnose MFS [8]. According to the revised Ghent nosology, a MFS clinical diagnosis is made based on scoring of systemic features, family/genetic history, aortic diameter, and the fibrillin 1 (*FBN1*) gene mutation status. However, the broad spectrum of anomalies exhibits variable expressivity, making this disease difficult to classify and diagnose in some cases. Thus, a genetic test is necessary to confirm a diagnosis.

The molecular basis of MFS is causal variants in the FBN1 gene. The FBN1 protein is a structural macromolecule, which contributes to the integrity and function of all connective tissues and forms fibers visible under electron microscope [9]. FBN1 not only has a structural role as an important component of the microfibrils but also has a role in the sequestration and activation of the growth factor TGF-*β*. The insufficient expression of the *FBN1* gene leads to severe rupture of the elastic fiber network structure, followed by aortic dilatation and hardening of the wall. Thus far, more than 1,500 variants in the *FBN1* gene have been identified [10, 11]. Copy number variants (CNVs) account for less than 10% of cases with MFS [12]. Despite significant advances in recent years, the genetic basis of CNVs for MFS remains elusive.

Benke and Agg [12] reported a case of a 32-year-old woman with MFS harboring a 31956bp *FBN1* microdeletion by whole-genome sequencing (WGS). This small microdeletion was also present in the patient's sister and mother. Prakash et al. [13] reported that a recurrent rare genomic duplication in 17q25.1 was related to the aortic wall lesions in early onset TAAs and dissections.

Exploring the underlying genetic etiology of CNVs in MFS patients may provide more information about diagnosis, such as CNV length and affected genes. This genetic data may also prove to be an invaluable source for understanding how haploinsufficient genes contribute to disease pathogenesis.

In this study, we report a hereditary TAA pedigree with a novel 0.76 Mb microdeletion in 15q21.1. This pathogenetic CNV, leading to haploinsufficiency of the *FBN1* gene, cosegregates with the disease phenotype in the family.

## 2. Materials and Methods

### 2.1. Study Subjects

We analyzed a TAA pedigree (two females and one male) with suspected MFS from the Genetic Counseling Clinic and the Department of Cardiovascular Surgery, the First Affiliated Hospital of Jinan University, China, in March 2020. All the patients underwent a complete examination according to the revised Ghent nosology 2010 for MFS.

This study protocol was approved by the Research Ethics Committee of the First Affiliated Hospital, Jinan University. Written informed consent was obtained for all family members. They agreed to a comprehensive physical examination and genetic research. Family histories were obtained by interviewing the family members. Available clinical data, including medical records, electrocardiograms, and computed tomographic angiography (CTA), were systematically reviewed. Approximately 2.0 mL of peripheral venous blood was collected, and DNA was extracted with the Gentra Puregene Blood Kit (QIAGEN, Santa Clara, CA, USA) in accordance with the manufacturer's instructions.

### 2.2. WES and Variant Analysis

To systematically search for disease-causing gene mutations, exome sequencing in three affected individuals, II-2, II-3, and III-3, and three normal individuals, I-1, II-4, and III-4, was performed. Genomic DNA samples from affected patients and healthy members were obtained with written informed consent. Genomic DNA was extracted from peripheral blood samples of the family members using the QIAamp DNA Blood Mini Kit according to the manufacturer's instructions (Qiagen GmbH, Hilden, Germany). Exome capture and high-throughput sequencing (HTS) was performed by the Illumina HiSeq2000 platform. Five micrograms of genomic DNA from three affected individuals, II-2, II-3, and III-3, and three normal individuals, I-1, II-4, and III-4, were captured with the Agilent SureSelect Human All Exon V5 Kit (Agilent Technologies, Inc., Santa Clara, CA, USA), according to the manufacturer's protocols.

WES reads of 300 bp fragments were mapped. An average sequencing depth of 100× was reached, and >99% variants of the genome were covered at least 20×. The annotated variants were screened with databases, including the single nucleotide polymorphisms SNP database (https://www.ncbi.nlm.nih.gov/projects/SNP/), GenomeAD (https://gnomad.broadinstitute.org/), and 1000 Genomes Project (1000G, https://1000genomes.org). The pathogenetic variants were estimated in accordance with the American College of Medical Genetics and Genomics (ACMG) technical standards of CNVs.

The resulting qualified reads were submitted to an in-house bioinformatics pipeline and then were aligned to the reference human genome (hs37d5) using the Burrows-Wheeler Aligner. We analyzed data with Chromosome Analysis Suite (ChAS) software (Affymetrix).

A joint consensus recommendation of the ACMG and the Clinical Genome Resource (ClinGen) for technical standards of constitutional CNVs was published in 2019. Namely, a laboratory geneticist should assign any CNV reported in a patient to one of the five main classification categories: pathogenic (P), likely pathogenic (LP), variants of uncertain significance (VUS), likely benign (LB), and benign [14]. Only genes functioning in a dominant manner that are within the pathogenic CNVs and likely pathogenic CNVs were investigated in this study. All annotated CNVs were experimentally validated by real‐time quantitative PCR (qPCR). During the initial period of the study, two patient samples tested by fluorescence in situ hybridization (FISH) were also detected by CMA.

### 2.3. Chromosomal Microarray Testing and CNV Evaluation and Validation

To confirm our WES results, we performed chromosomal microarray testing using the CytoScan HD chip (Affymetrix, Santa Clara, CA, USA) in accordance with the manufacturer's instructions. Moreover, we analyzed data with ChAS software (Affymetrix), which had a calling threshold of 20 consecutive probes encompassing at least 25 kb in length. Genomic DNA was extracted from the peripheral blood samples. The first strand of cDNA was synthesized with this DNA sample as a template and designed primers. Then, the cDNA fragments were amplified by PCR with the primers, labeled, and hybridized. We analyzed pathogenic CNVs using Agilent Cytogenomics software (Agilent Technologies).

All the reported CNVs were subject to the build 37 of human genome/hg19 on NCBI. The screened CNVs for comparative analysis had to meet the following conditions: (1) deletions ≥50 kb/25 markers; duplications ≥100 kb/50 markers; (2) <50% overlap with known segmental duplications (SD); and (3) not found in the control populations cataloged in the Database of Genomic Variants (DGV). We selected 178 individuals without heart disease from our local database as controls. Other controls were selected from the SNP database (https://www.ncbi.nlm.nih.gov/projects/SNP/), 1000 Genomes Project (1000G, https://1000genomes.org), and the DGV (https://dgv.tcag.ca/dgv/app/home).

## 3. Results

### 3.1. Patient Demographic and Clinical Characteristics

**Figure 1:**
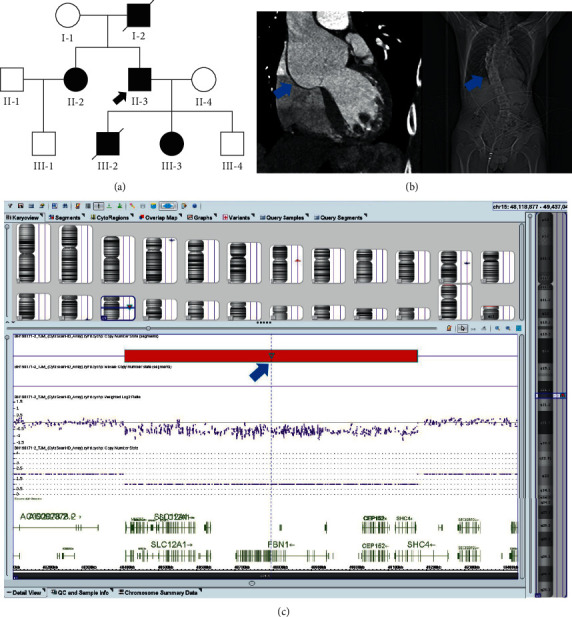
Investigation of 15q21.1 microdeletion pathogenicity. (a) Pedigree of family carrying the heterozygous 15q21.1 microdeletion. Full and open circles and squares indicate MFS patients and normal females and males, respectively. Patient II-2 was diagnosed with MFS and atrial septal defect. The proband is marked by a black arrow. Patients I-2 and III-2 with diagonal lines represent dead. Patients II-2, II-3, and III-3 carried the heterozygous CNV. (b) The blue arrows show the proband presenting with aortic aneurysm (left) on a CTA of the thoracic aorta and scoliosis (right) of the thoracolumbar spine on a radiograph. (c) The pathogenetic 15q21.1 microdeletion confirmed by CMA. In the blue frame is chromosome 15, and the red bar shows the deletion area. The deletion cite of CNV is Chr 15: 45,087,159–52,465,173.

A three-generation family pedigree exhibiting TAA (Figure 1(a)) was obtained. All the enrolled and affected family members had previously been diagnosed with TAA or aortic root dilation at the time of enrollment.

Two generations in the family exhibited an autosomal dominant pattern, and their clinical manifestations, including aortic aneurysm and scoliosis, both met two major diagnostic criteria in the revised Ghent nosology for MFS.

#### 3.1.1. Patient II-3

In the family pedigree (Figure 1(a)), the proband was patient II-3. The patient II -3 was a 33-year-old man. His weight was 61 kg; height was 180 cm (90–97^th^ percentile); and arm span was 180 cm. He had an aortic aneurysm, and radiography revealed scoliosis of the thoracolumbar spine (Figure 1(b)).

#### 3.1.2. Patient III-3

Patient III-3, a 9-year-old girl, was the proband's daughter and the second child of the affected father. Her birth weight was 3200 g (25–50^th^ percentile) with unknown birth height. She completed developmental milestones in time and excelled at sports in school. Her body weight was 25 kg (3^rd^ percentile); her height was 140 cm (90–97^th^ percentile); and her arm span was 140 cm. She did not have myopia. Echocardiography showed mitral valve prolapse (MVP) and aortic root dilation (sinus of Valsalva: 3.3 cm (Z score: 3), sino-tubular junction (STJ): 1.90 cm). Radiography revealed no scoliosis of the thoracolumbar spine.

#### 3.1.3. Other Family Members

The proband's father died suddenly at the age of 56 due to aortic rupture. Before death, he had symptoms of acute chest pain without obvious inducement. The proband's first son died of cardiovascular defects at the age of one year. The proband's sister, patient II-2, had aortic root dilation (expanded diameter 3.6 cm) and minor atrial septal defect (ASD) that did not require closure. Notably, the proband's second son, patient III-4, was healthy with no suspected signs and symptoms of MFS.

### 3.2. Exome Sequencing Analysis and Segregation of Variants

Exome sequencing of six individuals (II-2, II-3, III-3, I-1, II-4, and III-4) generated 73,230,394 pairs of sequenced reads in total, with an average depth of 129.89×. In total, 93.50% of the sequenced reads passed the quality assessment. The qualified reads were mapped to 99.37% of the human reference genome.

The family was also examined by WES to identify the causative gene associated with TAA. However, after filtering, no candidate gene variants for the phenotypes could be identified using our typical WES methods. Subsequently, we decided to determine putative CNVs using WES read coverage. Using our screening strategy, a series of microdeletions in 15q21.1 (48 460 852–48 779 693) × 1, (48 780 317–49 037 302) × 1, and (49 060 309–49 168 811) × 1 were observed in the proband.

### 3.3. CMA Identification of a Pathogenic CNV and Cosegregation Analysis

CMA conﬁrmed the deletion detected by WES and revealed a 0.76 Mb microdeletion in 15q21.1 (Chr15:45 087 159–52 465 173; Figure 1(c)) in the patients II-2, II-3, and III-3. This microdeletion is very rare but overlapped with well‐characterized MFS CNVs (DECIPHER database). This microdeletion encompasses *FBN1*, a gene known to play an important role in aortic development or structure/function [15]. Besides *FBN1,* eight additional genes were located in the region, including solute carrier family (*SLC24A5*) 24 member 5, (*MYEF2*) myelin expression factor 2, (*CTXN2*) cortexin 2, (*SLC12A1*) solute carrier family 12 member 1, (*DUT*) deoxyuridine triphosphatase, (*CEP152*) centrosomal protein 152, (*SHC4*) SHC adaptor protein 4, and (*EID1*) EP300 interacting inhibitor of differentiation 1.

The microdeletion in 15q21.1 was absent in the healthy individuals (II-4 and III-4). As such, the heterozygous 15q21.1 microdeletion completely cosegregated with MFS in the family. The genetic information for patient I-2 was unavailable. Additionally, CNVs identified in two patients (II-3 and III-3) were listed in the Database of Genomic Variants and were considered to be likely pathogenic.

Taken together, WES and CMA results indicate that Del 15q21.1 CNV may contribute to the pathogenesis of MFS in this family.

## 4. Discussion

In this study, we applied WES and effective filtering of a TAA pedigree and identified a series of pathogenic CNVs in 15q21.1. We confirmed the CNV using CMA and demonstrated that this pathogenetic CNV cosegregated with MFS in this family.

The atypical MFS proband only suffered from TAA, scoliosis, and myopia (250°) and did not present with ectopia lentis or other classic clinical manifestations. In the revised Ghent nosology, the standards of scoring for each systemic feature of MFS are listed, such as wrist and thumb sign (scoring three points), myopia >3 diopters (scoring one point), scoliosis (scoring one point), and mitral valve prolapse (scoring one point). According to the revised Ghent nosology, if the proband has a family history, a systematic score of 7 points is sufficient for a clinical diagnosis of MFS. Our proband's clinical manifestations only scored two points, which did not meet the diagnostic criteria of MFS [8]. However, the genetic result confirmed the molecular cause of the disease and helped us diagnose the patients as MFS. For the proband's affected child, we recommended improving the lifestyle and having regular follow-up visits twice per year. The affected daughter already had mitral valve prolapse and aortic root dilation; given that aortic dimensions were small and/or the rate of aortic dilation was slow, we suggested annual echocardiography to monitor the status of the ascending aorta. If the rates of aortic dilation exceed approximately 0.5 cm per year, she should accept more frequent examinations such as computed tomography (CT) or MR angiography (MRA). In daily life, the girl needs to avoid strenuous exercise to prevent joint damage and avoid agents that cause vasoconstriction. Our genetic diagnosis is an important supplement to the differential diagnosis of the proband, which can differentiate MFS from Shprintzen–Goldberg syndrome (OMIM: 609460), Loeys–Dietz syndrome (OMIM: 609192), and vascular Ehlers–Danlos syndrome (OMIM: 130050). These diseases have similar clinical features but are caused by mutations in different genes. In addition, we also provided genetic information of the proband's offspring and confirmed that the CNV was familial and heritable.

Here, we identified a recurrent rare CNV that overlapped with the MFS critical region; the size of the deleted fragment was 0.76 Mb. The deletion region encompassed nine genes, including the FBN1 gene, which is related to cardiovascular development. Of note, haploinsufficiency of *FBN1* is an important pathological mechanism in TAA and aneurysmal dilation of MFS, which may be explained by decreased tension and elasticity of the aortic wall [15].

*FBN1* controls the phenotype by modulating the TGF-*β* signaling pathway. *FBN1* deletion leads to fibrin 1 deficiency resulting in TGF-*β* activation, TGF-*β* signal overexpression, and increased free TGF-*β* level [16,17]. The other eight genes are generally not associated with cardiovascular lesions. For example, *SLC24A5*, *MYEF2*, and *CTXN2* gene variations have been associated with differences in skin pigmentation, partial albinism, and retinal pigment; *SLC12A1* plays a key role in concentrating urine, and its defects result in some Bartter-like syndromes. *DUT* gene encodes an essential enzyme of nucleotide metabolism; its overexpression leads to extensive DNA excision and repair, resulting in DNA division and cell death. Mutations in *CEP152* have been associated with primary microcephaly; *SHC4* is mainly expressed in the testes and brain; and *EID1* may play a crucial role in lipid accumulation and proliferation of neural stem cells. Further studies of these genes on the normal allele are pending. All our patients had no other clinical manifestations than those that can be attributed to the deletion of *FBN1*.

Thus far, in a few cases, the deletion of the whole *FBN1* gene has been confirmed through molecular cytogenetic techniques. Faivre et al. [18] reported a patient with a 2.97 Mb 15q21.1-q21.2 microdeletion (hg18 chr15:45 459 710:48 427 149) associated with MFS. The deletion contained 13 OMIM genes. The patient had flat feet, speech delay, a mitral insufficiency with a dystrophic valve, and T6–L1 evolutive scoliosis with a maximal curvature of 21°. Another study detailed four large genomic rearrangements in *FBN1* [19] and revealed that deletions between exons 24 and 53 of *FBN1* tended to cause more severe clinical phenotypes than haploinsufficiency or the *FBN1* pathogenic variant alone. For example, patients, with a deletion of exons 49 and 50, had severe neonatal MFS phenotypes such as distal airspace enlargement, frequently resulting in spontaneous lung rupture. In contrast, patients with deletions of exons 24–26, exons 33–38, and exon 30 showed the most severe MFS phenotypes, including abnormal head, long anteroposterior diameter, ugly face, increased skin wrinkles at brow center when crying, high arch and narrow palatal arch, small pupil, spider finger, and excessive joint activity. It has also been found that different exon deletions of *FBN1* may be associated with different clinical manifestations of MFS [19]. To the best of our knowledge, this current report is the first description of the smallest known microdeletion containing the *FBN1* gene. There are currently few reports on CNVs of MFS [12, 13, 18, 19], and additional CNVs still remain to be explored. The relationship between the MFS phenotypes that are atypical or mild and CNVs also deserves further research.

Today, WES is widely used in laboratory tests for MFS diagnosis. Compared with traditional FISH screening, WES is not only suitable for identifying typical variations and CNVs of classical MFS, but it can also identify other potential atypical variations and CNVs for nonclassical MFS, due to its high resolution and high accuracy at the whole-genome level. Furthermore, CMA is suitable for the study of CNVs due to its high resolution and throughput. Taken together, our results suggest that if WES is combined with CMA, accurate and comprehensive CNV results can be achieved.

The present study has some limitations. First, the visual development of these MFS patients could not be accurately assessed because of medical conditions and the young age of the children. Second, we did not have information about CNVs for all the probands' parents and first son. Thus, only some genetic information related to CNVs was obtained.

## 5. Conclusions

We identified a novel 15q21.1 microdeletion (Chr15:45 087 159–52 465 173) in a Chinese Han family with suspected MFS by WES and CMA. The CNV encompasses *FBN1*, which plays an important role in classic MFS. These findings enrich the available phenotypic observations of MFS with CNVs of the *FBN1* gene, and 0.76 Mb CNV may be the smallest known microdeletion. These data can help patients gain access to more accurate genetic counseling to make informed decisions for their future.

## Data Availability

All data generated or analyzed during this study are included in this article.

## References

[1] Judge D. P., Dietz H. C. (2005). Marfan’s syndrome. *The Lancet*.

[2] Zhao S., Duan Y., Huang F., Shi Q., Liu Q., Zhou Y. (2020). A novel splicing mutation in marfan syndrome. *International Journal of Legal Medicine*.

[3] Wu Y., Sun H., Wang J. (2020). Marfan syndrome: whole-exome sequencing reveals de novo mutations, second gene and genotype–phenotype correlations in the Chinese population. *Bioscience Reports*.

[4] Rurali E., Perrucci G. L., Pilato C. A. (2018). Precise therapy for thoracic aortic aneurysm in marfan syndrome: a puzzle nearing its solution. *Progress in Cardiovascular Diseases*.

[5] Mercier F., Fabiani J. (1998). Surgery of thoracic aortic aneurysms: modern surgical treatment and results. *Journal of Cardiovascular Surgery*.

[6] Vanem T. T., Rand-Hendriksen S., Brunborg C., Geiran O. R., Røe C. (2020). Health-related quality of life in marfan syndrome: a 10 year follow-up. *Health and Quality of Life Outcomes*.

[7] De Paepe A., Devereux R. B., Dietz H. C., Hennekam R. C. M., Pyeritz R. E. (1996). Revised diagnostic criteria for the marfan syndrome. *American Journal of Medical Genetics*.

[8] Loeys B. L., Dietz H. C., Braverman A. C. (2010). The revised ghent nosology for the marfan syndrome. *Journal of Medical Genetics*.

[9] Pyeritz R. E. (2017). Etiology and pathogenesis of the marfan syndrome: current understanding. *Annals of Cardiothoracic Surgery*.

[10] Kayhan G., Ergun M. A., Ergun S. G., Kula S., Percin F. E. (2018). Identification of three novelfbn1mutations and their phenotypic relationship of marfan syndrome. *Genetic Testing and Molecular Biomarkers*.

[11] Collod-Béroud G., Le Bourdelles S., Ades L. (2003). Update of the UMD-FBN1mutation database and creation of anFBN1polymorphism database. *Human Mutation*.

[12] Benke K., Ágg B., Meienberg J. (2018). Hungarian marfan family with large FBN1 deletion calls attention to copy number variation detection in the current NGS era. *Journal of Thoracic Disease*.

[13] Prakash S., Kuang S.-Q., Regalado E., Guo D., Milewicz D. (2016). Recurrent rare genomic copy number variants and bicuspid aortic valve are enriched in early onset thoracic aortic aneurysms and dissections. *PLoS One*.

[14] Riggs E. R., Andersen E. F., Cherry A. M. (2020). Technical standards for the interpretation and reporting of constitutional copy-number variants: a joint consensus recommendation of the American college of medical genetics and genomics (ACMG) and the clinical genome resource (ClinGen). *Genetics in Medicine*.

[15] Gülan U., Calen C., Duru F., Holzner M. (2018). Blood flow patterns and pressure loss in the ascending aorta: a comparative study on physiological and aneurysmal conditions. *Journal of Biomechanics*.

[16] Cook J. R., Clayton N. P., Carta L. (2015). Dimorphic effects of transforming growth factor-*β* Signaling during aortic aneurysm progression in mice suggest a combinatorial therapy for marfan syndrome. *Arteriosclerosis, Thrombosis, and Vascular Biology*.

[17] Lindsay M. E., Dietz H. C. (2011). Lessons on the pathogenesis of aneurysm from heritable conditions. *Nature*.

[18] Faivre L., Van Kien P. K., Callier P. (2010). De novo 15q21.1q21.2 deletion identified through FBN1 MLPA and refined by 244K array-CGH in a female teenager with incomplete marfan syndrome. *European Journal of Medical Genetics*.

[19] Li J., Wu W., Lu C. (2017). Gross deletions in FBN1 results in variable phenotypes of marfan syndrome. *Clinica Chimica Acta*.

